# Gut Microbiota and Their Role in Health and Metabolic Disease of Dairy Cow

**DOI:** 10.3389/fnut.2021.701511

**Published:** 2021-08-04

**Authors:** Qingbiao Xu, Qinqin Qiao, Ya Gao, Jinxiu Hou, Mingyang Hu, Yufeng Du, Ke Zhao, Xiang Li

**Affiliations:** ^1^College of Animal Sciences and Technology, Huazhong Agricultural University, Wuhan, China; ^2^College of Information Engineering, Fuyang Normal University, Fuyang, China; ^3^Institute of Food Science, Zhejiang Academy of Agricultural Sciences, Hangzhou, China; ^4^National Center for International Research on Animal Genetics, Breeding and Reproduction (NCIRAGBR), Huazhong Agricultural University, Wuhan, China; ^5^Key Laboratory of Agricultural Animal Genetics, Breeding and Reproduction of Ministry of Education, College of Animal Sciences and Technology, Huazhong Agricultural University, Wuhan, China

**Keywords:** gastrointestinal microflora, metabolic diseases, rumen, ruminants, dairy cow, omics

## Abstract

Ruminants are mostly herbivorous animals that employ rumen fermentation for the digestion of feed materials, including dairy cows. Ruminants consume plant fibre as their regular diet, but lack the machinery for their digestion. For this reason, ruminants maintain a symbiotic relation with microorganisms that are capable of producing enzymes to degrade plant polymers. Various species of microflora including bacteria, protozoa, fungi, archaea, and bacteriophages are hosted at distinct concentrations for accomplishing complete digestion. The ingested feed is digested at a defined stratum. The polysaccharic plant fibrils are degraded by cellulolytic bacteria, and the substrate formed is acted upon by other bacteria. This sequential degradative mechanism forms the base of complete digestion as well as harvesting energy from the ingested feed. The composition of microbiota readily gets tuned to the changes in the feed habits of the dairy cow. The overall energy production as well as digestion is decided by the intactness of the resident communal flora. Disturbances in the homogeneity gastrointestinal microflora has severe effects on the digestive system and various other organs. This disharmony in communal relationship also causes various metabolic disorders. The dominance of methanogens sometimes lead to bloating, and high sugar feed culminates in ruminal acidosis. Likewise, disruptive microfloral constitution also ignites reticuloperitonitis, ulcers, diarrhoea, etc. The role of symbiotic microflora in the occurrence and progress of a few important metabolic diseases are discussed in this review. Future studies in multiomics provides platform to determine the physiological and phenotypical upgradation of dairy cow for milk production.

## Introduction

Nearly 200 species of ruminants were identified till date, and among them, six were domesticated (1). Dairy cow was the most studied. Earlier studies provide insights into the knowledge of their digestive metabolism. Ruminants (mostly herbivores) employ foregut fermentation that allows them to digest cellulosic materials from plants. But during evolution, vertebrates lost the ability to produce enzymes that degrade cellulose and other complex polysaccharides (2). The ruminants rely upon a symbiotic relationship with microorganisms to digest such compounds. The microbiota produces enzymes to break the complex compounds into simpler molecules for easy absorption by the intestine. To carry out this, the host system has to provide an optimal environment and substrate for the survival of microflora. Thus, a commensal relationship is maintained where the host organism provides the substrates and maintains the environment required for the survival of the organism. In return, the microflora offers the nutrients required for the host organism (3).

**Figure 1:**
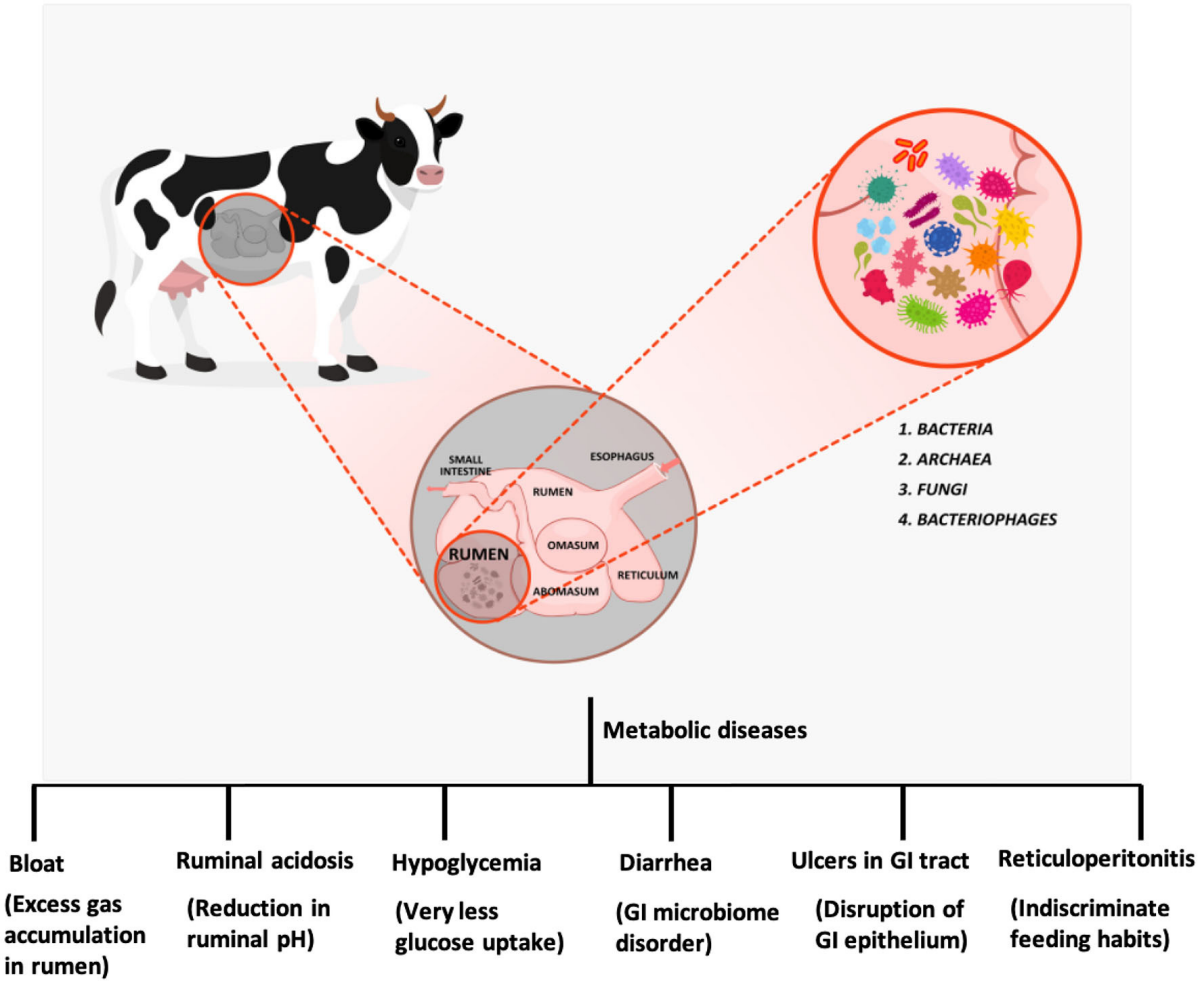
Ruminant digestive system, microflora and metabolic diseases in ruminants.

The physiology and structure of the ruminant digestive system evolved billion years ago to ensure the effective digestion of cellulosic materials and various polysaccharides (4). The potency of the system lies in its design where the ingested feed material experiences a prolonged interaction with microflora (5). The ruminant stomach is a quadra compartmental digestive sac composed of the rumen, reticulum, omasum, and abomasum. Rumen internal environment is partitioned into different sacs by reticulo-ruminal fold in which the ingested food enters the rumen and then the reticulum (Figure 1). The rumen is lined with papillae, whereas the reticular epithelium forms a honeycomb structure. Feed consumed is directed toward the rumen through the reticulum (6). Reticulorumen (collective chamber of rumen and reticulum) stores the feed consumed for rumination and interaction with microflora. The feed is chewed to mix it with saliva and then swallowed. The ingested feed is then transferred to the anterior reticulorumen. Saliva is crucial for ingestion as well as rumination. It contains phosphate, potassium, and sodium bicarbonate in high concentrations to buffer the acids generated during fermentation. The reticulorumen appears to be a multifunctional fermentation sac with sizes varying from cattle (35–100 L) and sheep (3–5 L) (7). The physicochemical parameters of the rumen are described in Table 1 (9–13). The host organism maintains the environment of rumen through various mechanisms. The atmosphere in the reticulorumen is mostly anaerobic with carbon dioxide (65%), methane (27%), nitrogen (7%), and hydrogen (0.2%) (14, 15). Along with these, traces of O_2_, H_2_S, and CO are also present. This gas composition is due to the rigorous fermentation in the rumen by resident microflora. The ingested feed is regurgitated to facilitate proper fermentation through interaction with microflora, a process called rumination.

**Table 1:** Physicochemical properties of the rumen.

**Parameter**		**References**
Temperature	39°C (optimal), vary in between 38–41°C	(2, 8)
pH	~6.5 (buffered in the range of 5.5–7.0)	
Dry content	Maintained constant around 10–20%	
Osmolality	250–400 mOsmol/Kg (increases with the feed intake)	
Redox potential	Lies within the range of −150 to −350 mv	
Gaseous composition	CO_2_ (65%), CH_4_ (27%) are the major gases produced by fermentation. N_2_ (7%), O_2_ (0.6%) H_2_ (0.2%) are present in traces.	

Rumination helps in increasing the surface area and decreasing the size of the feed particles, thereby promoting proper fermentation (16–18). In continuation, after the degradation of feed particles into smaller compounds, the feed is passed into the following chamber omasum. Omasum plays the role of a filter through which lesser size (<2 mm) particles can freely pass through (19). Then the digested fodder moves to abomasum, the true stomach. The abomasum has a distinct enzyme component lysozyme that attacks the cell walls of bacteria (20, 21). In abomasum, the digestion of bacterial proteins as well as digesta is done in a similar fashion as the other non-ruminants (17). Host genetics also play a crucial role in deciding the fate and constitution of rumen microflora, which in turn has an effect on fermentation and the products (22). The maintenance of the rumen environment is crucial for the host to digest the feed and survive (23). This process in turn effects the ability of the dairy cattle to produce milk. The constitution of microflora is very important for all the above reasons. Deviation of the constitution of microflora or intrusion of infective organisms through feed, environmental, and other factors leads to disturbances in the metabolism of the host. This leads to diseases, and the regulation of such process is mandatory. Present review throws light on the roles of gut microbes in the health and metabolic diseases of ruminants.

## What Is the Need of Microflora?

Ruminants feed on plants that are the sources of complex polysaccharides, *viz*., hemicellulose, cellulose, and lignin (24). However, due to the lack enzymatic system to degrade polysaccharides, they employ microflora that are capable of hydrolyzing these compounds in the gut for energy generation (25). These commensal microbes utilize the feed consumed by the host for survival, thereby establishing a healthy symbiotic relationship. The microbial population is habituated in the reticulorumen compartment. The reticulorumen environment is strictly anaerobic. It comprises dense and diverse microflora with eukarya (fungi and protozoa), archaea (methanogens), and bacteria at concentrations of 10^4^, 10^6^, and 10^10^, respectively (26). These bacterial populations seem to be very sensitive and can be influenced by little changes in the rumen environment. Fermentation by the rumen microflora is a complex process in which microorganisms act in coordination to generate simpler compounds that are easily metabolized by the host (27). The polysaccharides are metabolized into two simpler forms. The former one is the proteins required for bacterial cell wall synthesis and the later form is the volatile fatty acid (VFA), which are end products of fermentation (28, 29). VFA plays a crucial role in ruminant metabolism and acts as a source of host energy. They participate in vital pathways such as fatty acid synthesis and gluconeogenesis. Recent findings indicate that VFA holds ~70% of plant polysaccharides energy content (30, 31).

## Members of Microbial Consortium

Primarily rumen is hosted by various microorganisms that assist the host organism in the digestion of complex polysaccharides of the dairy cow. The infants derive basal flora from the environment, feed consumed, partners, etc. The early gut microflora is developed from breast feeding (43%) and environment (28%), whereas non-breastfed lambs receive from vagina (46%) and air (31%) (32). The first floor of the rumen of neonates is colonized by enterococcus and streptococcus species which transform the gut environment to anaerobic (33). This helps in the recruitment of strict anaerobes in the gut to maintain the anaerobic ambience. Facultative anaerobes and aerobes are present in very less quantities, approximately 100-fold lesser in comparison with anaerobic organisms. The digestive capability of the dairy cow is directly proportional to the existing rumen microflora activity. Most of the organisms present in the rumen are non-culturable, whereas the culturable biota were studied in all aspects (34, 35).

The constitution of gut microbiota varies with the host, indicating a solid environmental-driven specificity of the host. The microbial composition of the feces in twins was more similar than in siblings (36). This implies the involvement of host genetics in deciding the individual gut microflora. Individuals also vary in fungal and archaeal compositions. The choice and development of gut microflora hence is a collaborative play of host genetics as well as environment. It is an ardent fact that the physiology of the individual has a strong relationship with gut microbial development. Apart from this, microflora differs from section to section in gut regions. Strict segregation of microflora between digestive and epithelium starts in the early stages of the life of a calf. The methanogenic composition also differs down the gastrointestinal tract. In neonatal calves the phylum *Bacteroides* is predominant, whereas in adult animals the phyla *Prevotella* and *Bacteroidetes* are abundant. Studies indicate that the microflora of 21-day-old calves has *Prevotella* (15.1%) and *Bacteroidetes* (15.8%), implying a starter-feed-driven rumen microbiome development during maturation. Methanogens and cellulolytic members were observed at 3–4 days of age, and this population is similar to that of matured mammals. Cellulolytic flora is present in 1-day individuals, indicating their importance in the ruminant system. Surprisingly, the rumen microflora of 14-day-old calves harbors more profuse yet ephemeral microorganisms in comparison with adult organisms. Metagenomic studies indicate that archaea (0.6–4%), eukarya (1.5%), and bacteria were present in ascending order of magnitude, with bacteria contributing 95% of the coding sequence.

### Bacteria

Bacteria occupies the major portion of gut microflora, and their presence is crucial for the health of the dairy cow. They aid in the fermentation and degradation of plant polymers by the secretion of various enzymes (37, 38). Rumen contains about 1 × 10^3^ bacteria/mL, and this consortium is complex in terms of functionality and taxon identification. The communal interaction of various bacteria enables the breakdown of the ingested fiber. The identification of these bacteria and their unique functionality has become the focal point of many studies. With the advancement of next generation sequencing technology, microbiological techniques, culture free approaches, and genetic engineering it has become easier to step forward in studying the role of commensal flora in host metabolism. Gene sequencing helped to identify and classify bacteria based on 16 s rRNA and physicochemical properties. The predominant microflora in the rumen are *Proteobacteria, Bacteroidetes*, and *Firmicutes*. In a study, *Prevotella* has also been identified with 42–60% of rRNA composition in two lactating cows. The coordinated metabolism of the microflora in which the metabolic product of one organism acts as a substrate for the other allows the sequential digestion of plant polymers (39, 40). The bacterial consortium is highly complex, and hence most of the bacteria are uncultured. The flora which are dominant and have a specific role in the metabolism are covered here so far.

The cell wall of a plant is comprised of a hemicellulose matrix with embedded cellulose fibers in it. The initial degradation of this matrix is carried out by a particular taxon of bacteria that secretes cellulolytic enzymes (24, 25). In general, bacteria contribute to most of the xylanase and endoglucanase activities in the rumen. These degrade the cellulose into smaller oligo/disaccharides which are then acted upon by other organisms. The first order cellulolytic bacteria includes *Ruminococcus flavefaciens, Ruminococcus albus*, and *Fibrobacter succinogenes*. Also, *Butyrivibrio fibrisolvens* is present in a lesser extent in comparison with the above said organisms. Apart from these, other uncultured bacteria can also act upon the substrate to degrade cellulose fibers. Some organisms like *Cellulosilyticum ruminicola H1*, from the rumen of Yak, also have the capability to produce lignocellulolytic enzymes. On the other hand, coculturing of some organisms implicated negative interaction and decreased enzyme efficiency (41). This inhibition is found to be an effect of the bacteriocins secreted as a part of the defense mechanism and competition for the substrate (42). For instance, *R. flavefaciens* and *R. albus* secrete bacteriocins in competition for cellulose (43, 44). Non-cellulolytic bacteria also secrete bacteriocins and are supposed to be tough competitors for different substrates in a rumen environment (45, 46).

The end products of cellulolytic bacterial interaction act as substrates for different microflora that start further degradation of such compounds. Other important polymers, such as starch, are hydrolyzed by *Selenomonas ruminantium, Succinomonas amylolytica, Butyrivibrio fibrisolvens, Streptococcus bovis, Ruminobacter amylophilus*, and *Prevotella* species, whereas pectin is degraded by *Lachnospira multiparus* and *Succinovibro dextrinosolvens*. Besides, the constitution of bacteria changes with the type of feed consumed by the host (5, 47, 48). Animal feeding differs in various places. High fiber feed is rich in cellulose whereas high grain feed is packed with starchy material. This influences the type of bacteria required to digest the material consumed and has a strong impact on microflora constitution in the gut environment (49, 50). Sugar and starch fermenters constitute most of the rumen bacteria. Maximum energy is extracted from the plant polysaccharides as the end products of bacterial fermentation serve as substrate to many other organisms. *Megasphaera elsdenii* acts upon lactate (end product of bacterial fermentation) and *Veillonella alcalescens* utilizes succinate, acetate, and hydrogen (51, 52).

Recent metagenomic studies on gut microflora of various mammalian species revealed that in ruminant and herbivore microflora the anabolic pathways for the synthesis of amino acids (AAs) are more prevalent in comparison to carnivores. This is because the diet of a carnivore would be rich in protein, and therefore the constitution of gut microbiota is chosen to be more proteolytic. In the point of herbivores, the diet is fiber rich, and carbohydrate is the core source of energy (53). Hence in the microbiota of rumen, the AA synthesis pathways are commonly seen. Indeed, a certain cellulolytic activity some organisms also exhibit potent proteolytic activity, such as *B. fibrisolvens, P. ruminicola, S. ruminantium*, and *R. amylophilus. P. ruminicola* exhibits deaminase and proteolytic activities and produces higher amounts of ammonia (NH_3_) in the rumen. This activity is considered to be crucial as the rumen environment has lesser protein and ammonia that act as nitrogen sources for AA and protein synthesis (54). Other classes of bacteria include sulfate-reducing bacteria that assist in the reduction of sulfate to H_2_S. In addition, it has to be noted that the rumen microbiota is fine-tuned depending upon the dietary changes to assist degradation and fermentation of various complex compounds. They also have communal relations with each other and with the host to ensure their survival as well as maximum energy production. They also play a role in supplying VFAs and proteins to the host organism. Disturbances in concentrations of microbiota sometimes have a heavy impact on the host system and may lead to diseases. Different types of bacteria are listed in Table 2.

**Table 2:** Gut bacteria in ruminants (mostly rumen).

**Bacteria type**	**Bacterial species**	**Gram staining**	**End products**	**References**
Cellulolytic	*Fibrobacter succinogens*	Negative	Acetate, Formate, Ethanol, propionate	(55–60)
	*Ruminococcus flavefaciens*	Positive		
	*Ruminococcus albus*	Positive		
	*Clostridium longisporum*	Positive		
	*Eubacterium cellulosolvens*	Positive		
	*Clostridium cellobioparum*	Positive		
	*Butyrivibrio fibrisolvens*	Negative		
Hemi cellulolytic	*Eubacterium xylanophilum*	Positive	Acetate, Formate, Ethanol, propionate	(55–60)
	*Eubacterium uniformis*	Positive		
	*Prevotella ruminicola*	Negative		
Lipolytic	*Anaerovibrio lipolytica*	Negative	Acetate and propionate	(61)
Pectinolytic	*Treponema saccharophilum*,	Negative	Acetate and formate	(62)
	*Lachnospira multiparus*	Positive		
Proteolytic	*Prevotella sp*.	Negative	Amino acids, nitrogen	(63)
	*Ruminobacter amylophilus*,	Positive		
	*Clostridium bifermentans*	Positive		
Amylolytic	*Prevotella ruminicola*	Negative	Formate, propionateand Acetate	(64)
	*Streptococcus Bovis*,	Positive		
	*Ruminobacter amylophilus*	Positive		
Saccharolytic	*Succinivibrio sp*.	Negative	Lactate, Acetate, Fumarate, Succinate	(55–60)
	*Lactobacillus sp*.	Positive		
	*Bifidobacterium ruminantium*	Positive		
Tanninolytic	*Streptococcus Caprinus*	Positive	Lactate, Acetate, Fumarate, Succinate	(62)
	*Eubacterium oxidoreducens*	Positive		
Ureolytic	*Megasphaera elsdenii*	Negative	Ammonia and CO_2_	(55–60)

### Archaea

Anaerobic methanogens make up most of the archaea constituting ~0.6–3.3% of the total rumen microbiota (65). Major archaea members of rumen microbiota are listed in Table 3. Metagenomic studies and 16 s rRNA sequencing analyses revealed the presence of archaea in the rumen environment. Studies revealed that about 3.6% of microbiota in rumen exhibited autofluorescence, a distinctive property exhibited by methanogenic bacteria (71). Methanogens, as the name indicates, generate methane (CH_4_) either by the reduction of CO_2_ or by the hydrolysis of acetate to CH_4_ and CO_2_. Most of the ruminal methane is produced via the reduction of CO_2_ rather than dissimilating acetate. The process of CO_2_ reduction requires electrons which come from various sources, including methylamine, methanol, formate, and hydrogen produced as metabolic intermediates (72, 73). Archaea are clustered under Euryarchaeota and are classified as *Methanomicrobiales, Methanosarcinales, Methanococcales, Methanobacteriales*, and *Methanopyrales*. Most of the ruminant methanogens fall under one of the three categories identified. They are ordered as *Methnaomicrobiales* < *Methnaomicrobium* and *Methanobacteriales* (14.9%) < *Methanobrevibacter* (61.6%). Apart from this, another set of uncultured ruminal archaea were categorized under rumen cluster C (RCC). A study on the ruminal archaea community of red deer, cattle, and sheep disclosed the fact that their composition is maintained throughout different species. They are more conserved when compared to the bacterial members. The dominant archaea species stood same in all the rumens. Species belonging to *Methanobrevibacter* is found to be dominant in rumen. About 26.5% of the total archaea is occupied by members of RCC (55, 66).

**Table 3:** Various microflora in rumen.

**Organism**	**Species**	**Mode of action**	**References**
Archaea	*Methanobacterium formicicum, Methanobacterium bryantii, Methanobrevibacter ruminantium, Methanobrevibacter smithii, Methanomicrobium mobile, Methanosarcina barkeri, Methanoculleus olentangyi*	Strictly anaerobic and produce methane from CO_2_ and H_2._	(65, 66)
Protozoa	*Entodinium bovis, Entodinium bubalum, Entodinium bursa, Entodinium caudatum, Entodinium chatterjeei, Entodinium parvum, Entodinium longinucleatum, Entodinium dubardi, Entodinium exiguum, Epidinium caudatum, Isotricha prostoma, Isotricha intestinalis, Dasytricha ruminantium, Diplodinium dendatum, Diplodinium indicum, Oligoisotricha bubali, Polyplastron multivesiculatum, Eremoplastron asiaticus, Eremoplastron bubalus*	Lignocellulosic digestion and degradation of complex compounds to reducing sugars	(67)
Bacteriophages	*Methanobacterium phage Ψ M1, Methanobacterium phage Ψ M10, Methanobacterium phage Ψ M100, Methanothermobacter phage Ψ M100, Methanobacterium phage ΨM2*	Strictly anaerobic and produce methane from CO_2_ and H_2._	(3)
Fungus	*Piromyces communis, Piromyces mae, Piromyces minutus, Piromyces dumbonicus, Piromyces rhizinflatus, Piromyces spiralis, Piromyces citronii, Piromyces polycephalus, Anaeromyces mucronatus, Anaeromyces elegans, Caecomyces communis, Caecomyces equi, Caecomyces sympodialis, Cyllamyces aberensis, Cyllamyces icaris, Neocallimastix frontalis, Neocallimastix patriciarum, Neocallimastix hurleyensis, Neocallimastix variabilis, Orpinomyces joynii, Orpinomyces intercalaris*	Act upon lignin and cellulose fibers to and forms Formate, Succinate, Hydrogen, acetate and lactate.	(68–70)

Methane production by various archaea is mediated by cytochrome in few methanogens, whereas alternative complexes mediate this process in some methanogens. The genus *Methanosarcinales* comprises of methanogens and has the capability to grow on a wide range of substrates. Hydrogen concentration in the environment plays a crucial role in the production of methane. Cytochrome-based methanogens have higher growth yields when compared with non-cytochromic methanogens. Non-cytochromic methanogens need lesser hydrogen concentration to produce methane whereas cytochromic methanogens need about 10-fold higher concentrations of hydrogen for the optimal growth. This is the reason for the presence of non-cytochromic methanogens in higher concentrations in the rumen. Hydrogen utilization by methanogens is crucial as it decreases the pressure, allowing the conversion of endergonic metabolic reactions to exergonic reactions. This makes bacterial fermentation energetically favorable (74). Hydrogen consumption by methanogens stands as a good example of the symbiotic relationship between methanogenic and cellulolytic bacteria, wherein the hydrogen produced by the latter is consumed by the former for its survival. Coculturing of rumen methanogens and ruminal fungus has a heavy influence on cellulolytic and fermentation activities. Hydrogen transfer among methanogens and other microflora in rumen is best described by coculturing methanogens with protozoa. Even though archaea and bacteria fall prey to protozoa, methanogens get habituated inside and help in the generation of energy by consuming the hydrogen produced during the metabolism (74–76). Hydrogen consumption by methanogens forms the root of symbiosis with other microbiota in the rumen for maximal energy production (77–79). The commensal interactions of methanogens with protozoa and other rumen microbiota facilitate the complete degradation of complex plant polymers. The methane production is directly related to the amount of fodder and hemicellulose degradation (80–82). About 19% of the total energy of the feed is lost during the production of methane gas by methanogens. The commensal interaction of methanogens with other microbiota in the rumen enhances energy production to a maximum extent. But the gas production has a hinderance effect on the overall energy harvested from the ingested feed.

### Protozoa

Protozoa are unicellular organisms bound by pellicle or cuticle in the rumen. They are the simplest forms of eukaryotes found in the universe (Table 3). Most of the protozoa are parasitic as they feed on microorganisms, organic matter, and cell debris. Ciliates are more prevalent in ruminant gut in comparison with several flagellate species. Ciliates are subcategorized into Vestibuliferida and Entodiniomorphida with 25 genera. Protozoa in the rumen have specialized functions tuned to survive in a rumen environment (83, 84). Most of the protozoa are anaerobic, but very few species are supposed to sequester oxygen. Oxygen sequestration from the environment is advantageous to the host as it maintains the anaerobic ambience of the reticulorumen. This also helps in the survival of strict anaerobes and promotes the digestive degradation. Various complex carbohydrates *viz*., lignocellulose, starch, and sugar are consumed by protozoa for energy production. Around 50% of the total biomass in the rumen is composed of protozoa. Degradation of fats, proteins, and carbohydrates is facilitated by direct engulfing (85). The lignocellulosic digestion capacity by protozoa is presumed to be the result of lateral gene transfer from the bacteria they engulf (86). Protozoa prey on selective species of bacteria, and the reason for feeding on particular bacteria is not clearly understood (87–89). Ciliates play a crucial role in fermentation and plant fiber degradation. The products obtained as a result of protozoan fermentation are found similar to that of bacteria. In contrast to bacteria, protozoa divide at a much slower rate (15–24 h). To overcome the washing out of protozoa before division, they tend to reside in the lower layers of the rumen. Many methanogens reside on the protozoan surface for H_2_. Hydrogen gas is produced is used for the reduction of CO_2_ to methane. Methanogens residing on protozoa account for around 9–25% of total rumen methane (77, 90). Protozoa are capable of engulfing and store more starch at once, which decreases acid production by lowering pH (91).

Protozoa (holotrich) produces pectin esterase, invertase, amylase, and polygalactouronase to degrade plant sugars and fibers. Protozoa also produce cellulolytic and hemicellulolytic bacteria in lower quantities compared with that of entodiniomorphids. Ciliates in the rumen secrete proteolytic enzymes, resulting in the production of AAs and ammonia. The type of engulfed microbiota decides the nitrogen metabolism of the protozoa. Generation of nitrogenous compounds in turn influences the recycling of nitrogen. Rumen ciliates also influence ammonia as well as VFA production. The symbiosis of protozoa and rumen bacteria were investigated and showed that the presence of rumen protozoa effected the bacterial composition in rumen. Absence of protozoa has a positive effect on the growth of cellulolytic and hemicellulolytic bacteria. Lambs with no protozoan population showed increased growth of wool as much as 10% when compared to lambs with rumen protozoa. No proper effect of protozoa on methane production is observed. Variations in the composition of digested material in both omasum and abomasum are observed in defaunated and faunated animals. It is an ardent fact that protozoa influence many processes in the metabolism of host (92–94).

### Fungi

Rumen is a repository of anaerobic fungi with an explicit capacity of lignocellulose degradation. Fungi contribute to 20% of the overall microbiota in the rumen. They are deliberate members of plant fiber degradation. Fungi also exhibit proteolytic activity. In the fungal structure, polycentric or monocentric thallus is observed, and the zoospores are polyflagellate or uniflagellate. Asexual life cycle of anaerobic fungi is mostly observed (95). Most of the fungi are not present alone in the rumen of the animals but are vividly present along the digestive tract. Fungal species were also isolated from the feces and saliva of the dairy cow. Domestic animals host Chytridiomycetes for assisting their digestion. These organisms occupy about 8% of total ruminal microbiota in the animals fed on forage, which allows more retention in the rumen (45). But in the case of high grain diets, fungal population decreases. Enzymes secreted by fungal cultures degrade lignin, hemicellulose, starch, and cellulose (33). In addition, fungi are strict anaerobes, and hence carbohydrate fermentation is the sole source of energy production. Fungi are devoid of cytochromes and mitochondria that are coplayers of oxidative phosphorylation. Despite that, they contain Hygrogenosomes that facilitate the generation of energy. Hydrogenosomes are mitochondrial derivatives that occurred during evolution, and they are not only confined to fungal genera. Various anaerobic eukaryotes and trichomonads are also found to contain this organelle. Hydrogenosomes differ from conventional mitochondria by possessing pyruvate/ferredoxin reductase instead of dehydrogenase. They also provide room for ATP production and pyruvate conversion.

Commensal interplay of fungi and bacteria is a well-studied concept. *In vitro* studies were carried out to understand the degradative dynamics of fungi when cocultured with cellulolytic bacteria. Cellulose degradation capacity of the fungi increases manifold with *Megasphera elsdenii, Selenomonas ruminantium*, and *Viellonella alcalescens*. Xylan consumption is increased by coculturing *Neocallimastix frontalis* with cellulolytic bacteria like *Selenomonas ruminantium, Prevotella ruminicola*, and *Succinivibrio dextrinosolvens* (Table 3). On the other hand, coculturing with *Streptococcus brevis* or *Lachnospira sp*. has a negative effect on xylan degradation. *R. flavefaciens*, and *R. albus* coculturing with fungi have shown adverse effects on cellulolytic activity. These bacteria release a polypeptide into the broth that has detrimental effects on cellulolytic activity of the fungus. The fungal activity in the degradation of cellulosic materials is considered minimum than that of bacteria. This might be due to their larger doubling time, inhibition by bacteria, competition for substrates, and decreased retention. Nevertheless, they exhibit remarkable activity in the degradation of lignocellulosic material, as the rhizoids pervade the cell wall of plants and make it easily accessible by the rest of the rumen microbiota (96).

### Bacteriophages

Bacteriophages are obligate parasites and play a crucial role in rumen microbiota. Bacteriophages infect bacteria and lyse them after their replication (Table 3). Through lysis, the overall bacterial population is maintained in the host digestive environment. Bacterial lysis releases bacterial proteins that act as precursors of AA synthesis (97). Bacteriophages are found to vary with the organism, i.e., they are specific for a particular organism. This may be used by the researchers to destroy a particular genus of microbes from the rumen environment. Very little information is known about the bacteriophages infecting protozoans, methanogens, and archaea. It was identified that siphophages are capable of infecting methanogenic bacteria. The knowledge about the enzymatic profile and genetic makeup of rumen phages is limited and yet to be explored to manipulate the rumen environment (98).

## Metabolic Disorders in Ruminants

Disturbances in the homogeneity of gastrointestinal microflora have severe effects on the digestive system and various organs. This disharmony in the communal relationship also causes various metabolic disorders, including bloat, ruminal acidosis, hypoglycemia, diarrhea, ulcers in gastrointestinal (GI) tract, and retivuloperitonitis (Figure 1).

### Bloat

The rumen tympany, also called as bloat, is associated with a condition in which excess gas is accumulated in the rumen. This is observed in animals fed with higher quantities of grains or forages (99), which can be categorized into free gas and frothy bloat. Free-gas bloat is associated with pathological/physical problems hindering gas release from the stomach of the dairy cow. Esophagus obstructions (external particles cloths and fruit material, etc.), cysts, blisters, tumors, thoracic or cervical enlargement, reticular dysfunction, and hypocalcemia are major conditions affecting gas belching (100–102). Frothy bloat is the result of feed ingestion, which continuously produces froth that cannot be easily expelled from the stomach. Testing with a stomach tube helps in figuring the type of bloat. If the causative agents are physical obstructions, they have to be removed manually to ensure the gas expulsion. Frothy bloat contains both hydrophobic and hydrophilic properties. The foam is the result of partial digestion of polymeric compounds including, lipopolysaccharides, fatty acids, glycans, and glycolipids. Presence of these partially digested compounds increases rumen viscosity and hinders gas removal. Gaseous distension exerts pressure on the nearby organs causing edema, pain, organ failure, and death. Several practices that are employed to treat free bloat and frothy bloat include using a stomach tube to remove gas and partially digested feed, anti-foaming agent administration, and the placement of fistula or cannula (103).

Apart from physical factors, the microbiota in the rumen also contribute to the development of gas. Gas is generated as a result of methanogenic bacterial action upon various substrates. This methane, hydrogen, and CO_2_ gases produced in excess when left unattended by downstream flora results in the accumulation of gas in the stomach. The hydrogen gas produced as a part of methanogen metabolism also has to be addressed. It is a well-known fact that the rumen environment is highly anaerobic. But excess CO_2_ can cause subtle changes in the rumen. CO_2_ can be reduced by methanogens to generate methane and/or as such CO_2_ in excess can cause tympany. It is nevertheless necessary to attend to the excess production of these gases to maintain ruminal microbial harmony. Hence to maintain the environment, probiotics can be used to replenish the rumen flora. Treated and high fiber feed also helps in relieving the stress caused by methanogenic bacteria (104).

### Ruminal Acidosis

Ruminal acidosis is caused by the consumption of more fermentable carbohydrate-rich feed material than grainy feeds (105, 106). Molasses, sugar beets, potatoes, and cereal grains result in acidosis. Fermentation of such compounds result in higher amounts of lactic acid production and hence pH of rumen is drastically reduced (107, 108). Due to this, many gram-negative bacteria are destroyed releasing endotoxin into the rumen. All these results in low pH, accumulation of fluid, disturbance of microbiota, and partial digestion. Low pH and acid production have destructive effects on the inner epithelium of the stomach causing ulcers as well as mucosal inflammation. Drastic fall in pH also inhibits the cellulolytic bacteria but enhances propionate-producing bacteria in the rumen. Rumen microbiota alteration leads to improper metabolism which can cause liver dysfunction, lung-related diseases, and can also lead to death (109–111).

### Hypoglycemia

Hypoglycemia is a disorder observed when the rate of glucose uptake is very less in comparison to the rate of utilization (112, 113). Vitamin B_12_ plays a key role in the synthesis of glucose from propionate, and its deficiency is also related to the occurrence of hypoglycemia. In new-born calves and lambs in a cold environment, hypoglycemia leads to death. Gluconeogenesis does require NADH and ATP apart from substrates made available in the ruminant environment. For this reason, an organism depends primarily on dietary carbohydrates for glucose rather than synthesis. Deficiency in glucose supply caused hypoglycemia in all the animals. On the other hand, hypoglycemia is also seen in animals whose diet is rich in inhibitors of fatty acid beta oxidation in the kidney and liver. Required amounts of AAs, fatty acids, ambience, and vitamins have to be provided for treating hypoglycemia (114–116).

Most of the fed polysaccharides should be degraded to glucose for energy production. Disharmony in the activity of rumen microbiota contributes to impaired degradation of polysaccharides that in turn affects glucose turnover. Proper diet at regular intervals with the maintenance of a favorable environment and supplementing cellulolytic bacteria may also address this issue in less severe conditions (117, 118).

### Ulcers in GI Tract

Ulcers in the dairy cow are more common in the duodenum and abomasum. They are often observed in cows and buffaloes than in sheep (119, 120). Ulcers are mostly associated with improper feed intake, over grazing stress, microbial infection, and malnutrition. These occur in concomitance with other diseases, *viz*., salmonellosis and blue tongue (*Clostridium perfringens* abomasitis). Over usage of non-steroidal antiinflammatory drugs can also cause ulcers. Perforating ulcers are generally more infectious and have adverse effects on the epithelium of gastrointestinal tract than non-perforating ulcers (121).

The disruption of the outer epithelium of gastrointestinal tract is caused by acid production and can be alleviated by the administration of probiotics containing lactic acid bacteria. Antihistamine with iron injection can also reduce the pain and bleeding in adult ruminants (122).

### Reticuloperitonitis

Reticuloperitonitis, also called as traumatic reticulitis or hardware disease, is mainly observed in cattle with unsystematic feeding (123, 124). Indiscriminate feeding habits of dairy cow leads to the disturbances in the harmony of rumen microbiota. Continuous feeding deters bacterial revival and causes improper digestion which may lead to bloat and ruminal acidosis. It is a noncontagious disease which if not properly observed causes devastating effects. Proper dietary consumption at regular intervals will enable bacterial resurgence and revival. Usage of probiotic syrups, administration of antibiotics, and digestive aids may help in the initial stages and rumenotomy is suggested during severity index (125).

### Diarrhea

Diarrhea is a severe problem prevalent in young calf. It is associated with various symptoms including disturbance of electrolyte balance, dehydration, and weakness. The reason for the disease varies with geographical location, type of feed, type of infection, and host metabolic issues. In most of the time, the disease occurrence is multifactorial. Pathogens namely, bacteria, virus, parasites, and protozoa can trigger infection. Infection by bacterial diarrhea includes *Enterobacter sp. mycobacterium paratuberculosis, Clostridium perfringens, Salmonella sp*. as well as *Staphylococcus*. Rotavirus and adenoviruses contribute to viral infections. *Trichonema sp*. and *Strongylus sp*. are major parasites infecting the gastrointestinal tract of the dairy cow. Nonetheless, *Trichomonas sp., Entamoeba sp.*, and *Giardia sp*. contribute to protozoan infection. Infection of the ruminant flora by either of the above species causes disturbance in the homogeneity and functionality, culminating in disease. Malabsorption or hypersecretion of fluids into the gut usually results in the secretion of excessive fluid from the intestine. Severe outflux of fluids with salts leads to weakness. Things to be observed to treat diarrhea are suppressing the infection and adjusting physiological imbalance. This allows eradication of the causative agent helping in faster recovery. Usage of antibiotic drugs will also help in wiping out the existing infection and maintaining the functional role of microflora (126).

## Role Of Multiomics in Dairy Cow

Gut microbiota plays a crucial role in ruminant digestion as well as energy production. Hence it is essential to study the genomic environment to predict the changes that cause genetic and metabolic disorders. But it is difficult to isolate and study the genome of a particular flora in the consortium. For this reason, the whole genome of the consortium is studied under the branch metagene “omics”. The complete genome of the gut microflora, termed as gut microbiome, is obtained by sequencing methodologies and omics approaches (127). The identity of the microbiome is determined by general sequencing protocols, whereas “omics” determine the actual functionality of the microbiome present in rumen. Omics approaches embrace metabolomics, metaproteomics, metatranscriptomics, and metagenomics. The relationship between host and microbiota is well-studied by omics approaches. For instance, metagenomics approaches revealed that *Bacteroidetes* is energetically less favorable to the host in comparison to *Firmicutes*. The action of *Firmicutes* increases nutrient availability to the host, which culminated in obesity.

Role of omics in the physiology and functionality of livestock is an area which is yet to be explored. Many omics-related approaches succeeded in finding the relation between microbiome composition and livestock production (128). These studies also helped in revealing taxonomical differences in the ruminal microenvironment of the organisms based on the dietary changes and environmental variations (22, 129). Recent studies on profiling microbiome of the rumen in a large sample set (>700) revealed a diet-dependent relationship between the host and microflora. The type of feed ingest decides the flora in the rumen (130). In depth analysis of the rumen microbiome using omics approaches helps in identifying markers that decide the variability in feed efficiency in cattle. Omics-based studies also help in assessing colonization patterns in the dairy cow.

## Conclusions and Future Perspectives

In the last few decades, the role of GI microbiota in health and disease has become the focal point of many studies. Involvement of gut microbiota in digestion and various diseases in humans is well-studied. However, in the case of dairy cows, the underlying mechanisms of host–microbial interactions are yet to be uncovered. The interaction of rumen or gut microflora is purely symbiotic in which one organism benefits the others. The higher organisms lost the capacity to degrade plant cell wall and other materials during evolution to use it as a source of energy. Hence, ruminants employed microorganisms to digest plant materials and in turn provided them nutrients required for survival. Several types of microorganisms reside in the rumen and gut of the dairy cow. These organisms are from all the main groups such as bacteria, protozoa, archaea fungi, and bacteriophages. Composition of rumen microbiota varies with the geographical location and type of regular feed. However, the dominant strains in the rumen environment are always conserved. Surprisingly, the microbiota adapts to the feed intake and changes its constitution to meet the requirement of the host. Bacteria occupy a major part of the ruminal microflora. Microorganisms are adopted in such a way that most of the energy is extracted from the provided substrate. Collaborative action of various species of organisms helps in proper digestion and energy production. The end product of one organism acts as a substrate for the secondary organism. In this manner, the degradation of the plant fiber is carried out to harvest maximum energy from the ingest.

To understand the metabolic disease of dairy cow, many factors have to be taken into consideration. This should start with the type of feed, interval of feed, grazing area, and response of the ruminant system to various drugs. Rumen microflora are the crucial role players in the digestion as well as energy generation for the dairy cow. Hence, it is nevertheless necessary for a dairy cow to maintain the ambience in the GI tract to ensure the proper symbiotic relationship with the resident bacteria. Infection by pathogens can lead to disharmony in the commensalism of the bacteria that culminates in various diseases. Prior identification of the infection, proper care, and treatment are required to rescue the organism. Preventive measures like proper ingest, probiotic supplementation, and vaccination protect the organisms from infections, thereby increasing the productivity. In depth analysis of microbiome using omics approaches helps in attaining knowledge about gut microbial mechanisms and functional activities at various conditions. Also, the variations in the gut microbiome have a strong impact on the phenotypic definition and physiology of the host. Gut microbiota has an influence on the health and productivity of dairy cow. Future studies in multiomics provide a platform to determine the physiological and phenotypical upgradation of the dairy cow for milk production.

## Author Contributions

QX and QQ wrote and prepared the original draft. YG, JH, MH, and YD edited the manuscript. QX, KZ, and XL critically reviewed the manuscript. All authors reviewed and approved the final manuscript.

## Conflict of Interest

The authors declare that the research was conducted in the absence of any commercial or financial relationships that could be construed as a potential conflict of interest.

## Publisher's Note

All claims expressed in this article are solely those of the authors and do not necessarily represent those of their affiliated organizations, or those of the publisher, the editors and the reviewers. Any product that may be evaluated in this article, or claim that may be made by its manufacturer, is not guaranteed or endorsed by the publisher.
